# Participation of Arterial Ischemia in Positional-Related Symptoms among Patients Referred for Thoracic Outlet Syndrome

**DOI:** 10.3390/jcm13133925

**Published:** 2024-07-04

**Authors:** Simon Lecoq, Mathieu Feuilloy, Pierre Abraham

**Affiliations:** 1 Service of Vascular Medicine, University Hospital, 49100 Angers, France; piabraham@chu-angers.fr; 2 Service of Sports Medicine, University Hospital, 49100 Angers, France; 3 INSERM, CNRS, MITOVASC, Equipe CarMe, SFR ICAT, University of Angers, 49100 Angers, France; mathieu.feuilloy@eseo.fr; 4 School of Electronics (ESEO), 49100 Angers, France

**Keywords:** thoracic outlet syndrome, transcutaneous oxygen pressure, pathophysiology, ischemia, upper limb, peripheral artery disease

## Abstract

**Objectives**: The coexistence of arterial compression with neurogenic thoracic outlet syndrome (TOS) is associated with a better post-surgical outcome. Forearm transcutaneous oxygen pressure (TcpO_2_) using the minimal decrease from rest of oxygen pressure (DROPmin) can provide an objective estimation of forearm ischemia in TOS. We hypothesized that a linear relationship exists between the prevalence of symptoms (PREVs) and DROPmin during 90° abduction external rotation (AER) provocative maneuvers. Thereafter, we aimed to estimate the proportion of TOS for which arterial participation is present. **Methods**: Starting in 2019, we simultaneously recorded forearm TcpO_2_ recordings (PF6000 Perimed^®^) and the presence/absence of ipsilateral symptoms during two consecutive 30 s AER maneuvers for all patients with suspected TOS. We retrospectively analyzed the relationship between the prevalence of symptoms and DROPmin results. We estimated the number of cases where ischemia likely played a role in the symptoms, assuming that the relationship should start from zero in the absence of ischemia and increase linearly to a plateau of 100% for the most severe ischemia. **Results**: We obtained 2560 TcpO_2_ results in 646 subjects (69% females). The correlation between PREVs and DROPmin was 0.443 (*p* < 0.001). From these results, we estimated the arterial participation in TOS symptoms to be 22.2% of our 1669 symptomatic upper limbs. **Conclusions**: TcpO_2_ appears to be an interesting tool to argue for an arterial role in symptoms in TOS. Arterial participation is frequent in TOS. Whether DROPmin could predict treatment outcomes better than the sole presence of compression is an interesting direction for the future.

## 1. Introduction

Asymptomatic thoracic outlet compression (TOC) can be observed in 23% to 62% of arterial investigations [1,2,3,4], while 67% of cadavers have abnormal anatomy at the thoracic outlet level [5]. Therefore, the association between upper limb symptoms and the presence of an arterial TOC may be coincidental. The standards report of the Society for Vascular Surgery for thoracic outlet syndrome has strongly underlined the interest in arguing for the presence of symptomatic ischemia in the upper limb to confirm that the relationship between symptoms and the presence of a positional arterial compression is not coincidental. To date, most thoracic outlet syndrome (TOS) is considered of neural origin (NTOS). Because it is stated that “symptomatic arm ischemia with arms elevated must be present for this diagnosis to be made”, this condition is considered a prerequisite for the diagnosis of arterial thoracic outlet syndrome (ATOS) [6]. Further, “Because the brachial plexus and subclavian artery traverse the same spaces, arterial signs and symptoms can be present in patients with NTOS although ATOS is not considered present unless proven symptomatic ischemia with compression or actual physical injury to the artery is present” [6]. As a result, to date, ATOS accounts for less than 3% of all TOS [7,8,9,10], but this does not exclude arterial participation in symptoms. As a matter of fact, the presence of arterial compression in neurogenic TOS seems associated with a better post-surgical outcome [11]. Beyond the presence of compression, its consequences in terms of ischemia are essential to evaluate. Transcutaneous oxygen pressure (TcpO_2_) recordings during dynamic maneuvers can be used to estimate the severity of exercise-induced ischemia [12,13,14,15,16,17]. Although a surface technique, it can provide evidence for the regional blood flow impairment at different levels and simultaneously on both sides. The relationship between symptoms and the severity of limb ischemia was previously studied in the lower limb [14] but not at the upper limb levels. 

We hypothesized that a significant relationship would be found between the prevalence of symptoms (paresthesia of the whole hand, upper limb positional pain, or fatigability) during provocative maneuvers and the severity of ischemia, as assessed with TcpO_2_ in patients with suspected TOS. Lastly, we aimed to estimate the proportion of tests where symptoms were likely to involve arterial participation in TOS.

## 2. Materials and Methods

### 2.1. Population and Ethical Standards

The SKIPA database is a longitudinal database of all adult patients (except if unable to understand the information for linguistic or cognitive reasons), referred to the Department of Vascular Medicine at the University Hospital of Angers for suspected TOS (Clinicaltrial.gov reference NCT04376177) starting January 2019. Patients were fully informed that they could deny the recording and use of their medical files for research purposes. We recorded the duration of symptoms, age, sex, weight, height, systolic and diastolic arm pressure, ongoing analgesic therapy treatments, the French version of the disease of the arm and shoulder (DASH) score [18], and results of available ultrasound investigations at the time of referral. As per routine, TcpO_2_ is one of the multiple tools that are used in our patients for the diagnosis of TOS. Most patients also had photoplethysmography, electromyogram, X-ray searching for a 13th or cervical rib, biological tests, etc., that are not reported here. At follow-up, only patients who were considered for surgery based on clinical and paraclinical observations had angiography (regular invasive arteriography). When these investigations were performed in our hospital, we were systematically informed of the results that were encoded in the database. As an observation of our medical routine and in accordance with French law, individual consent was not required. This study was performed as a retrospective analysis of this database until 1 January 2023 and complies with the principles outlined in the Declaration of Helsinki. It received local Ethics Committee approval under reference 2023/089.

### 2.2. Transcutaneous Oxygen Pressure (TcpO_2_) Recordings

TcpO_2_ recordings were performed systematically to evaluate the presence of arterial participation in symptoms and were performed blinded to the results of other investigations. We used a PF6000 (Perimed^®^, Jakobsberg, Sweden) with E5250 probes heated to 44 °C. Double calibration against air was performed before each recording session. Thereafter, we positioned probes on the dorsal aspect of each distal third of the forearms and on the trunk with the patient standing still or sitting. After 15 to 20 min of local heating, TcpO_2_ was recorded at 1 Hz to calculate decrease from rest of oxygen pressure (DROP) values (mmHg). Each recording was started 1 min before the first provocative maneuver for resting conditions. DROP corresponds to the subtraction of the chest TcpO_2_ changes from each of the upper limb TcpO_2_ changes from resting values [12,13]. The minimal DROP (DROPmin) during a provocative maneuver allows for eventual systemic arterial oxygen pressure (pO_2_) changes to be accounted for (such as hyperventilation or apnea due to pain during the provocative maneuvers) and is insensitive to the unpredictable transcutaneous gradient [17,19].

### 2.3. Provocative Maneuvers

**Figure 1:**
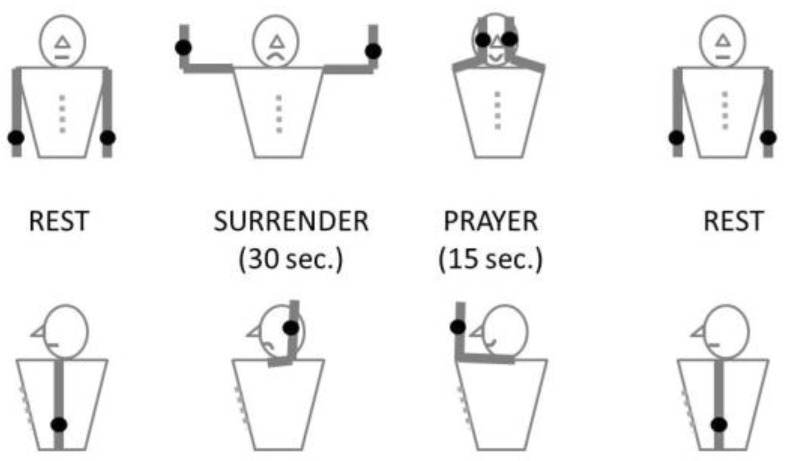
This is a schematic representation of the provocative maneuver used in our routine. The maneuver is an abduction external rotation (AER) test, Elevated Arm Stress Test (EAST), or Roos test, but the AER/surrender position is followed by a 15 s elevation without abduction (prayer position) to account for elevation only.

We used the abduction external rotation (AER)/Roos provocative maneuver [20], but after 30 s AER, this surrender (“Su”) position was changed to the prayer (“Pra”) position with arms elevated and elbows in front of the patient (Figure 1). This prayer position was maintained until second 45. Then, at second 45, the upper limbs were lowered. This Su-Pra sequence is of interest to normalize the duration of an eventual TOC. Note that this modification was developed for the recording and interpretation of venous investigations with photoplethysmography [20,21]. The procedure was repeated a second time for each patient when DROPs returned to zero after the first maneuver. For each maneuver, we recorded the presence (Symp+) or absence (Symp−) of positional symptoms of potential arterial origin (paresthesia of the whole hand, upper limb positional pain, or fatigability) during AER in each limb. 

### 2.4. Ultrasound and Angiography Results Encoding

The results of the ultrasound investigations were retrieved from the patient files. Ultrasound investigations were performed either by trained physicians in private practice laboratories or in the service of vascular Medicine at the University Hospital in Angers. From the report of the test, we recorded whether the presence of a “significant compression”, “>70% stenosis or compression”, or “an occlusive compression” was observed during the abduction maneuvers at the subclavian artery level on each side. Ultrasound was encoded on a limb-by-limb basis.

The results of arterial digital subtraction angiography were retrieved from the radiological report and classified for the presence or absence of arterial compression. Angiography was encoded positive if the presence of positional stenosis was “>70%” or referred to as “severe”, “pre-occlusive”, “significant or occlusive compression” of the subclavian artery at the costo-clavicular angle or inter-scalenic level. 

### 2.5. Data and Statistical Analysis

**Figure 2:**
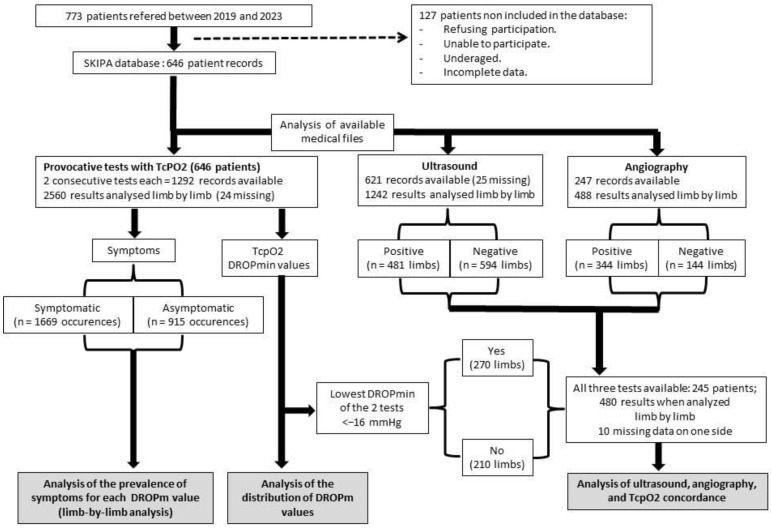
This is a summary of this study’s design and the three analyses that were carried out. TcPO_2_: transcutaneous oximetry; DROPm: minimal value of the decrease from rest of oxygen pressure index.

TcpO_2_ was encoded positive on a limb-by-limb basis for DROPmin < −16 mmHg [15,17]. The DASH disability/symptom score was calculated if 27 of the 30 items were available [18]. Results are reported as the number of observations (Nb) with percentage (%) of the observations within the considered group, mean ± standard deviation (SD), or median [25th–75th centiles], according to distribution normality. Distribution normality was tested using Kolmogorov–Smirnov tests. Note that the reliability of symptoms and DROPmin between test 1 and test 2 is presented in the additional data. For each DROPmin value, the prevalence of symptoms (PREVs) was calculated, on a limb-by-limb basis, and linear regression analysis was performed between these two variables. We anticipated that the number of expected DROPmin values (~60) was sufficient to determine a regression of r = or >0.40 with 80% power. Then, we first analyzed the relationship between PREVs and DROPmin values. Lastly, we determined the proportion of symptomatic cases that likely involved arterial participation (rounded to the unit), considering that in the absence of ischemia (DROPmin = 0 mmHg), the true probability that symptoms include arterial participation was null, and then it would increase linearly to 100% for the lowest DROPmin value (most severe ischemia). For all statistical tests, a two-tailed probability level of *p* < 0.050 was used to indicate statistical significance. Statistical analyses were performed using SPSS (IBM SPSS statistics V15.0; Chicago, IL, USA). A summary of the study design is presented in Figure 2.

## 3. Results

Of the 773 patients referred over the 4 years of the analysis, 127 patients were not eligible for data recording (refused recording or inability to understand the goal of this study) or were excluded because they were aged <18 years old or had incomplete TcpO_2_ data recording. We studied 646 subjects (age: 41.2 ± 11.6 years old; 69% females). Most patients (n = 393) complained of symptoms for more than 2 years, followed by 181 patients for more than six months, and the remaining 72 patients for less than six months. Almost all of these patients had already undergone multiple investigations before they were referred for suspected TOS. 

**Figure 3:**
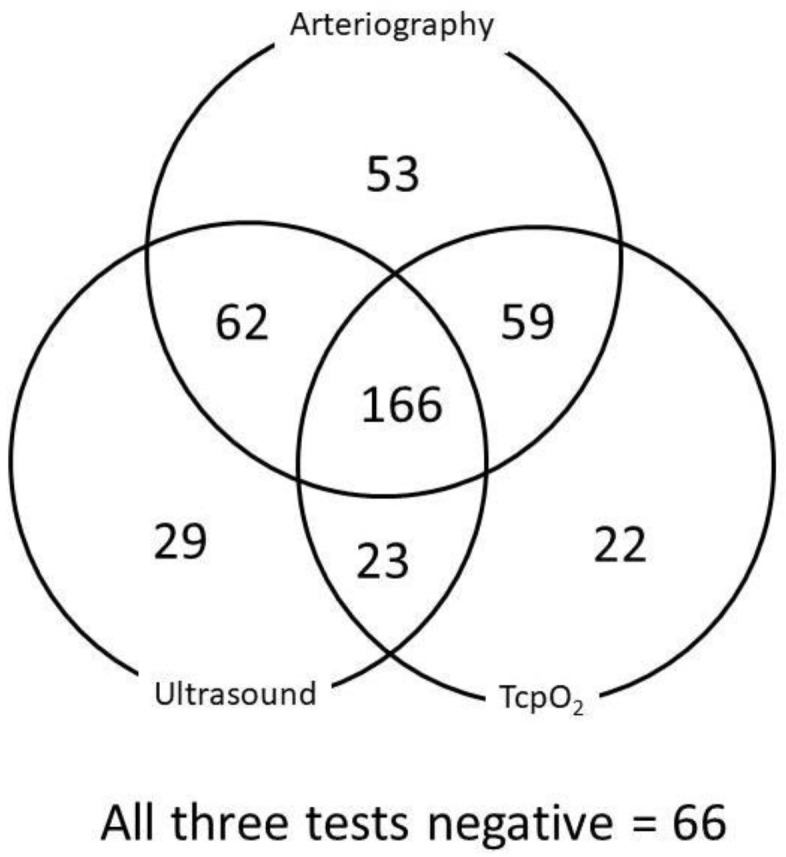
This figure shows the concordance among the results of angiography, ultrasound, and TcpO_2_ when all three tests were available for each case (n = 480). Results are expressed on a limb-by-limb analysis. The overlapping areas among the different circles represent the different cases when 1, 2, or 3 tests were positive at the same time (i.e., 166 limbs had all three exams concordant; 29 limbs had a positive ultrasound when TcpO_2_ and arteriography were negative; 59 limbs had positive arteriography and TcpO_2_ when ultrasound was negative). In a total of 66 cases, all three exams were negative.

Of the 621 available ultrasound results, 297 were negative, 68, 99, and 157 were positive on the right, left, and both sides, respectively. Most of the 25 missing ultrasound results either did not report the side of the investigation or were results from investigations performed before referral, which we were not able to obtain from the referring physician. Only 247 patients had angiography. Six of the patients were investigated on one side only or had no mention of the asymptomatic side on the radiological file, resulting in 488 results from 344 limbs encoded positive. Last, after encoding the lowest DROPmin value on a limb-by-limb basis to analyze the concordance of angiography, ultrasound, and TcpO_2_, we had 480 limbs in 245 patients with all three available results. Figure 3 shows the concordance of the three techniques on a limb-by-limb basis. Of interest is that perfect concordance among the three techniques was observed in less than half (48.3%) of the limbs with similar false positive and false negative results with ultrasound and TcpO_2_, if angiography is used as a gold standard. Finally, one patient had an arterial occlusion, and 26 (4%) were investigated for Paget Schroetter syndrome. Then, 95.8% of the patients were considered suspect of neurogenic TOS.

The DASH score was available for 521 patients and was 45.2 ± 20.3. Weight was 72.2 ± 16.6 kg (12 missing values), and height was 167 ± 9 cm (14 missing values). Half of the patients (52.8%) were under analgesic therapy for their symptoms. Among the included patients, 172, 160, and 305 subjects complained of positional symptoms by history (paresthesia of the whole hand, upper limb positional pain, or fatigability) on the right side, left side, and bilaterally, respectively. The other nine patients had missing information (n = 3) or were without apparent positional complaints at the time of referral following an episode of complication (venous thrombosis, n = 6). 

**Figure 4:**
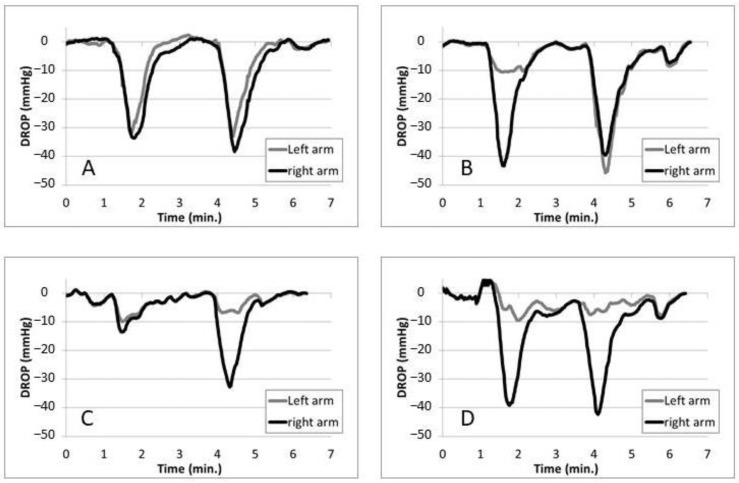
This figure shows typical transcutaneous oxygen pressure recordings expressed as a decrease from rest of oxygen pressure (DROP) during two consecutive Su-Pra maneuvers. (**A**) Patient A was a 44-year-old female and presented bilateral positional pain during the tests. She was lost on follow-up with a diagnosis of TOS. (**B**) Patient B was a 36-year-old female who complained of bilateral pain dominantly on the right side. Despite the absence of ischemia at the first test on the left side, the patient complained of pain on both sides and both tests. (**C**) Patient C was a 48-year-old male who was asymptomatic after treatment of unilateral TOS by first rib resection. He reported no positional pain during the tests. (**D**) Patient D was a 46-year-old male who reported unilateral right pain both by history and during the provocative maneuvers. Note that patients B and D had angiography that confirmed in both cases a positional occlusion at the costo-clavicular angle on the right side and severe non-occlusive stenosis on the left side.

A total of 1669 symptomatic occurrences were observed out of the 2584 measurements that were performed (two recordings for each arm of each subject). Absolute TcpO_2_ values at rest were 70.3 ± 12.7 mmHg, 72.1 ± 11.2 mmHg, and 70.8 ± 11.7 mmHg at the chest, right forearm, and left forearm, respectively. Of the expected 2584 DROPmin values, we had four and eight probe disconnections or technical failures on the right and left forearm, respectively, resulting in 24 missing DROPmin values. A typical example of DROPmin recording is presented in Figure 4. After the exclusion of these missing observations, we had 2560 results for DROPmin.

**Figure 5:**
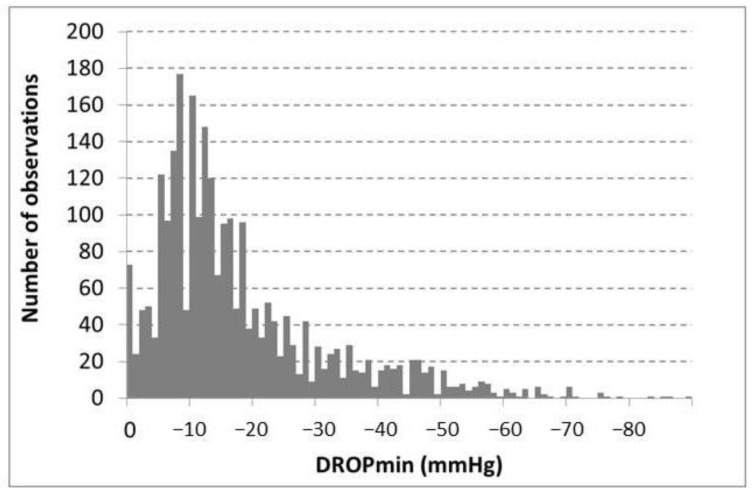
This figure shows the distribution of the minimal decrease from rest of oxygen pressure (DROPmin) values observed during the provocation maneuvers.

As shown in Figure 5, the lowest recorded DROPmin value was −89 mmHg. 

**Figure 6:**
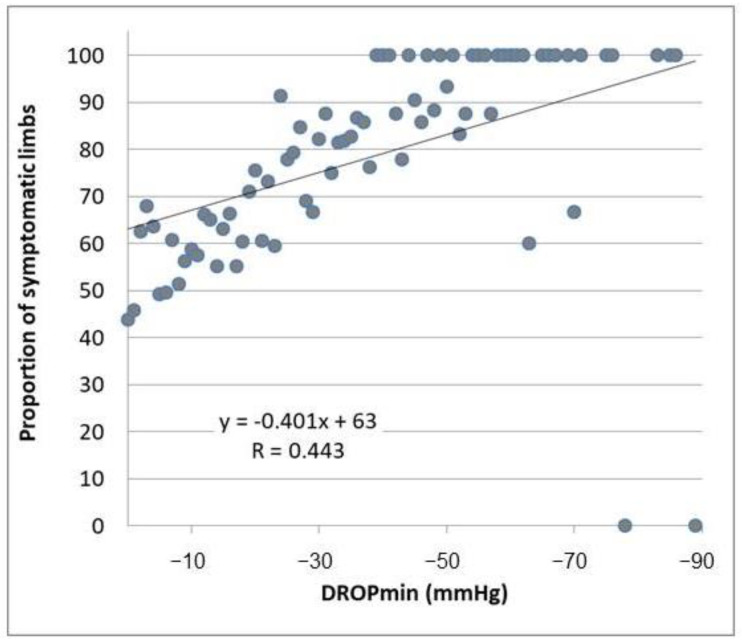
This figure shows a linear regression analysis of the relationships between the minimal decrease from rest of oxygen pressure (DROPmin) values and the prevalence of symptoms during the provocation maneuvers.

The “r” coefficient of correlation between DROPmin and PREVs over the whole range of DROPmin values was 0.443 (*p* < 0.001), confirming a moderate but significant relationship (Figure 6). Assuming that the true probability increases linearly from 0% for DROPmin = 0 mmHg to 100% for DROPmin = −89 mmHg by steps of 100/89 = 1.1236% for each unit of DROPmin decrease, and applying each probability to the number of symptomatic observations of each DROPmin value, we found 371 cases for which symptoms can—at least partly—result from coexisting arterial ischemia (22.2% of the symptomatic observations).

## 4. Discussion

As per our hypothesis, there was a significant relationship between DROPmin and the prevalence of symptoms in the upper limb of patients with suspected TOS, but many symptoms were not associated with coexisting ischemia. Advocating for the vascular origin of pain in patients with TOS is challenging [6]. Ultrasound allows simple, low-cost, and accurate recordings and remains relatively simple, but it cannot be performed strictly simultaneously on both limbs [3,22,23]. Indeed, even arterial attitudinal non-occlusive compression in the AER/Surrender position can provide sufficient perfusion to result in no significant oxygen delivery deficit. TcpO_2_ was reported to be insensitive to movements during lower limb treadmill tests and can be used during dynamic tests to provide quantitative results of regional blood flow impairment [13]. As for the lower limb, DROP calculation accounts for eventual systemic pO_2_ changes, which could result from increased/decreased arterial pO_2_ accompanying hyperventilation/apnea due to upper limb ischemic pain. 

There might be technical and clinical reasons for the low concordance among TcpO_2_, ultrasound, and angiography, which we also previously observed [15]. On the one hand, in ultrasound reports, results for positional tests are generally described in a binary way (presence or absence of compression) regardless of the type of test used, and the position of the patients (standing or lying). Differences in the positions of patients (lying vs. sitting), the time of each of the investigations, the procedures used to induce a positional compression, and the influence of the criteria used to define positive results should be considered when analyzing the concordance of the three techniques [24].

A significant relationship was found between the prevalence of symptoms and DROPmin values [14]. Since most of our patients were suspected to have neurogenic TOS, it is likely that most of the symptoms in upper limbs associated with DROPmin values close to zero were due to neural plexus compression or to pain resulting from non-TOS diseases. The latter hypothesis cannot be excluded, but it must be kept in mind that most patients referred to us had already undergone a prolonged and extensive diagnostic process before TOS was suspected. On the contrary, the high prevalence of coexisting ischemia in neurogenic TOS found in the present study is consistent with the prevalence of symptoms and positive arterial Doppler results observed in a small group of 22 patients by Likes et al. [11] and the prevalence of hand pallor arm discoloration during provocative maneuvers in neurogenic TOS [9,11,25]. 

There are limitations in the present work. Firstly, it could appear surprising that most DROPmin values range from 0 to −30 mmHg. We believe that this mainly results from altitudinal changes in the probe position relative to the heart (see Supplementary data for the analysis accounting for this effect). It could also result from non-occlusive artery compression from the limited duration of the AER/Surrender position (30 s) even in the case of complete arterial occlusion. Another study with symptom-limited AER would probably provide different results, but our aim was to standardize the duration of each test to facilitate comparisons among subjects.

Secondly, it could be suggested that other techniques to estimate tissue oxygen content (such as near-infra-red spectroscopy) would be preferable both because TcpO_2_ is a time-consuming technique and because spectroscopy claims to measure muscle oxygen content, although it is highly sensitive to skin flow changes [26,27]. This remains to be studied.

Thirdly, as a tertiary referral center, in our routine practice, we have limited information on the outcome and evolution of symptoms specifically for the patients that had efficient rehabilitation or negative investigations. Similarly, we have only very little information on nerve conduction studies or non-injected radiological imaging searching for bone anomalies in the database.

Fourthly, we report here only the results observed during AER, whereas many other provocation tests are proposed in the investigation of patients with suspected TOS, such as the Halstead maneuver, Wright’s, Cyriax Release, Allen’s test, and Adson’s tests. Their respective diagnostic performances are largely debated, and many discordant test results may be observed in the same patients [3,28]. 

Lastly, correlation with the characteristics of other clinical symptoms (shoulder pain, hand pallor, paresthesia, etc.) warrants future investigations. In our observations, patients frequently found it difficult to describe their symptoms, and this analysis was not performed. This point is important because the presence of symptoms in the limbs with DROPmin values remaining in the normal range could possibly result from isolated venous or neural TOC, as well as the possibility that they could result from osteo-articular symptoms not related to TOC. It is important to note that although we recorded positional-induced symptoms, osteo-tendineous lesions can also induce such symptomatology. Once again in most of our patients, multiple investigations had already been performed before referral, and TOS was rarely the first diagnostic hypothesis.

## 5. Conclusions

Using three different methodological approaches for detecting positional impaired inflow, less than half of the investigated limbs had consistent results, and many asymptomatic limbs showed positive results. From a clinical point of view, transcutaneous oximetry appears to be an interesting tool to fill the gap between the symptoms observed during provocative maneuvers and the presence/severity of ischemia. These results lead to the formulation of the concept that positional-induced ischemia may contribute to the development of symptoms in TOS; nevertheless, the results are not sufficient to fully explain the phenomenon, especially in neurological TOS. Future studies are needed to define the interest of TcpO_2_ in the diagnostic algorithm and the follow-up of patients with suspected TOS. Further, if the prevalence of arterial participation in TOS symptomatology is as high as we estimate, it casts doubt on some authors’ characterization of vascular investigations as useless [9,29]. 

## Data Availability

The datasets generated and/or analyzed during the current study are available from the corresponding author upon reasonable request.

## Supplementary Materials

The following supporting information can be downloaded at https://www.mdpi.com/article/10.3390/jcm13133925/s1. Reliability of transcutaneous oximetry and triphasic analysis of its relationship with the prevalence of symptoms. References [30,31,32,33,34,35] are cited in the supplementary materials.
