# Supplementary material for: Participation of Arterial Ischemia in Positional-Related Symptoms among Patients Referred for Thoracic Outlet Syndrome
Source: J Clin Med. 2024 Jul 4;13(13):3925. doi: 10.3390/jcm13133925 (PMC11242123; doi:10.3390/jcm13133925)

Reliability of transcutaneous oximetry and triphasic analysis of its relationship with the prevalence of symptoms.

BACKGROUND

Since the study used two consecutive tests, we aimed at analyzing the test-retest reliability of both symptoms reported and DROPmin results during the provocative maneuvers.

Further, in the determination of the relationship between the prevalence of symptoms (PREVs) of potential arterial participation (paresthesia of the whole hand, upper limb positional pain or fatigability) and the severity of ischemia as estimated from the decrease from rest of oxygen pressure (DROP), we aimed at accounting for the physiological changes in DROP that result from methodological issues. Indeed, in the absence of ischemia and gravity changes, the lowest DROP value throughout the test (DROPm) should remain close to zero during lower-limb exercise. Nevertheless, during TOS provocative maneuvers in the standing or sitting position, DROP shall decrease due to the changes in in probe position relative to heart level at the upper limb. Roughly the difference in probe position compared to heart level is 40 to 45 cm resulting in an expected physiological decrease in DROPm of down to - 9 or -10 mmHg [1].

Then, we hypothesized that the relationship between PREVs and DROPm should not necessarily be analyzed with a linear regression throughout the whole range of observed DROPm values but to a slightly more complex approach. We previously showed that at the lower limb level, PREVs increased with the severity of ischemia until a plateau would be reached for the most severe ischemia (lowest DROP values): diphasic relationship [2]. The difference from the lower limb is that at the upper limb, DROP is expected to decrease due to altitude change of the forearm probe relative to heart level during upper limb elevation. Then, at the upper limb level, DROPm values close to zero do not

indicate ischemia. If a linear relationship between PREVs and DROPm is to be observed at the upper limb during our maneuver, it should only start from a certain DROPm value (referred as DROPm1) that reflect the expected physiological decrease in DROP with hydrostatic pressure changes. As a result, the relationship of PREV and DROPm, at the upper limb was expected triphasic rather than diphasic. Indeed, as for the lower limb, following a linear relationship between PREVs and DROPm starting from DROPm1, a second inflexion point (DROPm2) would precede a plateau in the relationship of PREV and DROPm for values of DROPm lower DROPm2.

METHODS

Test –retest reliability

The Bland Altman representation was used to test the reliability of DROPm value observed during the first and second Su-Pra procedure. Kohen's Kappa calculation was used to estimate the agreement between symptoms observed between the two consecutive Su-Pra procedures, and interpreted according to Landis & Koch [3].

Symptoms and ischemia triphasic correlation

In order to objectively determine DROPm1 and DROPm2, we performed iterative determinations of the coefficients of correlation ("r") between DROPm and the PREVs starting from DROPm= 0 with a unit by unit increase in the number of DROPm (and then PREVs) values considered for the analysis. We expected that "r" for Pearson would start from 1 when only 2 DROPm values are used to calculate "r" (i.e; DROPm = 0 and DROPm = -1 mmHg) and would necessarily decrease with the increase in the number of DROPm (and then PREVs) values used to calculate "r", due to the absence of relationship between the DROPm and PREVs. Further increasing the number of DROPm (and then PREVs) values used to calculate "r", "r" would then increase in the interval where a linear relationship exists between DROPm and PREVs. Thereafter, still increasing the number of DROPm (and then PREVs) values used to calculate "r" until all DROPm values are used, "r" would decrease

when very low values of DROPm representing severe ischemia are involved in “r” calculation, because for lowest values of DROPm (most severe ischemia), a plateau is expected between DROPm and PREVs. We then determined DROPm1 and DROPm2 as the inflexion points of the relationship between “r” values and the range of DROPm values included for “r” calculation. DROPm1 and DROPm2 are the DROPm values resulting in the lowest and highest “r” values obtained by iterative “r” calculation, respectively.

Last we determined the proportion of symptomatic cases that were likely involving an arterial participation. Indeed, there is no reason why the probability that symptoms would –at least partly- result from underlying ischemia would abruptly change from 0 to 100%. For this purpose, we calculated the number (“N”) of DROPm values between DROPm1 and DROPm2. Thereafter, we considered the proportion of symptomatic cases likely involving an arterial participation as being zero from all DROPm values between 0 and DROPm1 (which consists in considering that all symptoms in this interval are not of arterial origin), as being 100% for values lower than DROPm2 (which consists in considering that all symptoms in these severe ischemic cases are of arterial origin) and as increasing by steps of “1/N” between DROPm1 and DROPm2. Then, we calculated the sum of all symptomatic cases including an arterial participation in our study.

RESULTS

Test –retest reliability

During the first Su-Pra procedure, 89 patients reported no symptoms, 131, 131, and 295 patients reported right, left and bilateral symptoms respectively. During the second Su-Pra procedure, 96 patients reported no symptoms, 141, 134, and 275 patients reported right, left and bilateral symptoms respectively. Although there was no systematic difference and a substantial agreement

(Cohen's Kappa 0.692+/-0.023) between symptoms observed during the two tests, as shown in table S1, symptoms reported during the two maneuvers were different in 21.5% of cases.

Median DROPm values were similar (-13 [-22;-8] mmHg) on the right side during both the first and second Su-Pra maneuver, and were -13 [-24;-8] mmHg and -12 [-23;-8] mmHg on the left side during the first and second Su-Pra maneuver, respectively. As shown in figure S1, DROPm values were relatively reliable between test 1 and test 2 with an average difference of 0.3 +/-8.8 mmHg.

Symptoms and ischemia triphasic correlation

The evolution of the "r" coefficient for the relationship between DROPm and PREVs with the increase in the number of DROPm values included in the analysis (from DROPm=0 to DROPm= - 98 mmHg) is shown in figure S2 (left panel).

As shown on the right panel, for DROPmin values ranging from 0 to -6 mmHg (absence of ischemia; n=447) the prevalence of pain (PREV) was already on the average 52.8%. Then, we found a linear increase in the prevalence of symptoms for DROPmin values (n=2081) ranging -7 to -61 mmHg (n=32). For DROPmin values lower than – 61 mmHg (most severe ischemia; n=32), the prevalence of symptoms plateaued (on the average 81.5%). In other words, this also means that on the average 460 (39.9%) of 1152 limbs with moderate ischemia (DROPm value from -7 mmHg to -16 mmHg) and 220 (22.9 %) of 961 limbs with DROPm <-16 mmHg (considered indicative of a significant ischemia) were asymptomatic during the provocation maneuver despite DROPm values considered indicative of ischemia.

Note that the “r” coefficient of correlation for the whole range of DROPm values reported in the manuscript was only 0.443 (p <0.001), but reached 0.878 (<0.001) when only values ranging from -7 to -61 mmHg were considered for the analysis.

In order to remove the symptomatic limbs that had no ischemia and were unlikely involving a vascular participation, and assuming that the true probability that the symptoms might –at least partly- result from coexisting ischemia was null for DROPm values equal or higher than -6 mmHg (i.e.; symptoms were unlikely of arterial origin), increases linearly from 0 to 100% between DROPm1 and DROPm2, by steps of 1/n = 1.852% for each unit of DROPm decrease then plateaued at 100% for DROPm < -61 mmHg, adapting the triphasic relationship to make it start from zero and plateau at 100%, and applying each probability to the number of symptomatic observations (n=1669) of each DROPm value, we found 381 cases for which symptoms can likely be considered as resulting –at least partly from coexisting ischemia (22.8 % of the observations with symptoms during the test, as shown in figure S3).

DISCUSSION

There may be clinical and technical reasons why test-retest agreement was substantial but not perfect for symptoms and showed consistency (mean close to zero) but still a significant standard error in the Bland-Altman agreement. Reliability of the elevated arm stress test was shown poor to good depending on which clinical parameter was tested [4]. We believe that the possibility that the second test was sensitized by the first one (as could result from insufficient recovery from the ischemia of the first test) was unlikely. Indeed, the number of symptoms observed during the two tests was almost similar, and the mean of differences in the Bland Altman plotting was close to zero. Another possible explanation relies on differences in the methods used for the tests. It was demonstrated that different maneuvers could provide different results in the same patients [5]. Although we standardized the durations of the AER position, small differences in the breathing pattern, in head, shoulder, elbow or hand positions may have influenced the test results.

Contrary to the lower limb where a diphasic relationship was observed starting from 0 mmHg, the problem during the upper-limb Su-Pra maneuver is the change in altitude of limb probes relative to the heart [2, 6], making values close to 0 mmHg resulting only from altitude change of the probes. It is of interest to account for the fact that the likelihood of an arterial participation to symptoms in the range of DROPm from 0 to -6 mmHg is expected zero and that the linear relationship starting from zero is not totally satisfactory. Inversely, although the observed PREVs was not 100% we believe that is very likely that symptoms always resulted from ischemia in the 26 cases of DROPm values < -61 mmHg associated to symptoms (1.02 % of all observations). One could suggest, that a formula could be applied to correct for the change in hydrostatic pressure. This is not what we did because the exact position of the probe on the forearm would depend on subject size, and because of the half-time response of TcpO2. Another solution to get rid of this problem would be to perform the tests in the lying position. This would make TcpO2 more comparable with the results of angiography. We were not keen to do so because it was previously shown that the prevalence of positive angiography

is higher in the sitting than in the lying position [7], and because usual symptoms are generally observed during community activities, sitting or standing [1, 8].

The fact that the proportion of symptoms for DROPm < -61mmHg was not 100% is very likely resulting from the fact that the duration of the AER/surrender position of Su-Pra maneuver was limited to 30 sec., which is possibly insufficient to induce symptoms. It is clear that normalizing the duration of the provocative maneuvers allows for a better inter-individual comparison, but it might have reduced the diagnostic performance of the test by limiting the decrease in DROP value resulting from impaired perfusion. Whether, at the individual level, the provocative maneuver should be as long as possible to enable better discrimination between the normal moderate DROP decrease due to gravity and abnormal DROP results, has yet to be determined.

Last, there is strictly no reason that the likelihood of symptoms to be –at least partly- of arterial origin would abruptly change from 0 to 100 % at -61 mmHg. Even considering that the determination of DROPm1 was underestimated and that all results between 0 mmHg and -12 mmHg should be normal results (normal decrease due to arm elevation relative to the heart) and recalculating with steps of 2% from DROPm = -12 mmHg to -61 mmHg, the total is still 321 of the 1669 symptomatic limbs during the test (19.2%).

CONCLUSION:

First, test-retest reliability of both symptoms and ischemia recorded during provocative maneuvers is acceptable but not perfect and suggest that maneuvers should probably be repeated rather than performed only once. Second, while the present analysis may appear very complex it seemed to us more satisfactory than the one reported in the manuscript. Nevertheless, the proportions of limbs for which ischemia was likely participating to symptoms (19.2% to 22.1%) resulting from this complex approach is only slightly lower than the one reported in the manuscript: 22.2%.

REFERENCES

1. Balderman J, Holzem K, Field BJ, et al. Associations between clinical diagnostic criteria and pretreatment patient-reported outcomes measures in a prospective observational cohort of patients with neurogenic thoracic outlet syndrome. *J Vasc Surg* 2017; 66: 533-544 e532. DOI: 10.1016/j.jvs.2017.03.419.
2. Blake DF, Young DA and Brown LH. Transcutaneous oximetry: variability in normal values for the upper and lower limb. *Diving Hyperb Med* 2018; 48: 2-9. DOI: 10.28920/dhm48.1.2-9.
3. Landis JR and Koch GG. The measurement of observer agreement for categorical data. *Biometrics* 1977; 33: 159-174.
4. Pesser N, de Bruijn BI, Goeteyn J, et al. Reliability and validity of the standardized elevated arm stress test in the diagnosis of neurogenic thoracic outlet syndrome. *J Vasc Surg* 2022; 76: 821-829 e821. DOI: 10.1016/j.jvs.2022.03.885.
5. Nord KM, Kapoor P, Fisher J, et al. False positive rate of thoracic outlet syndrome diagnostic maneuvers. *Electromyogr Clin Neurophysiol* 2008; 48: 67-74.
6. Blake DF, Young DA and Brown LH. Transcutaneous oximetry: normal values for the lower limb. *Diving Hyperb Med* 2014; 44: 146-153.
7. Cornelis F, Zuazo I, Bonnefoy O, et al. [Diagnosis of thoracic outlet syndrome. Value of angiography in the sitting position]. *J Radiol* 2008; 89: 47-52. DOI: 10.1016/s0221-0363(08)70369-x.
8. Povlsen S and Povlsen B. Diagnosing Thoracic Outlet Syndrome: Current Approaches and Future Directions. *Diagnostics (Basel)* 2018; 8. DOI: 10.3390/diagnostics8010021.

ADDITIONNAL FIGURES AND TABLES

Table S1: *Localization of symptoms (paresthesia of the whole hand, upper limb positional pain or fatigability) during the first (test 1) and second (Test 2) Su-Pra maneuvers.*

Test 1\Test 2	No symptoms	Right	Left	Bilateral	**TOTAL**
No symptoms	67	6	7	9	**89**
Right	5	110	1	15	**131**
Left	8	2	100	21	**131**
Bilateral	16	23	26	230	**295**
**TOTAL**	**96**	**141**	**134**	**275**	**646**

Figure S1*: Bland-Altmann representation of the results of DROPm observed on an arm-by-arm basis between the first and second Su-Pra procedure.*

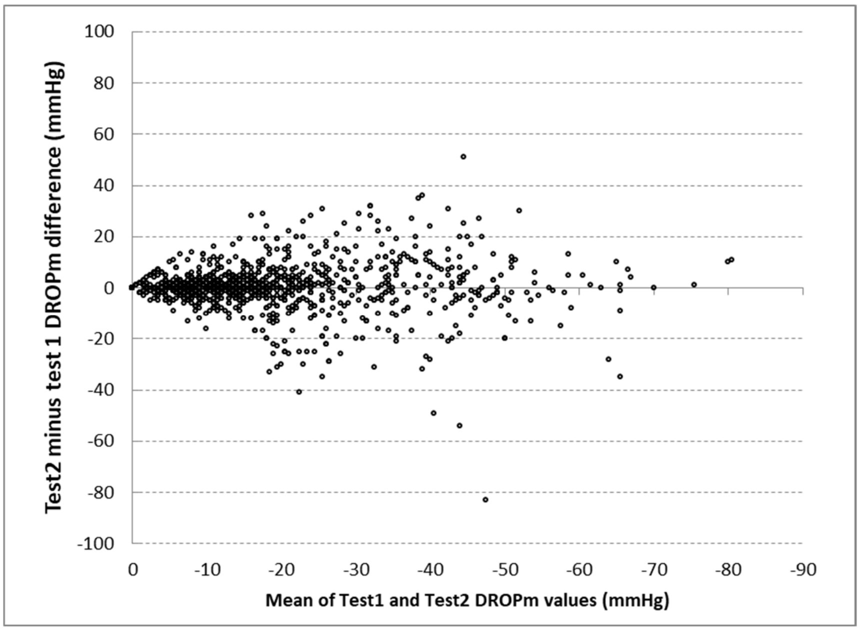

Figure S2: *Left panel is the evolution of the "r" coefficient of correlation with each increase in the number of DROPm values included in the analysis. The lowest and highest "r" values led to the determination of DROPm1 and DROPm2 respectively. On the right panel, the regression line is the one resulting from the analysis within the DROPm1- DROPm2 range only.*

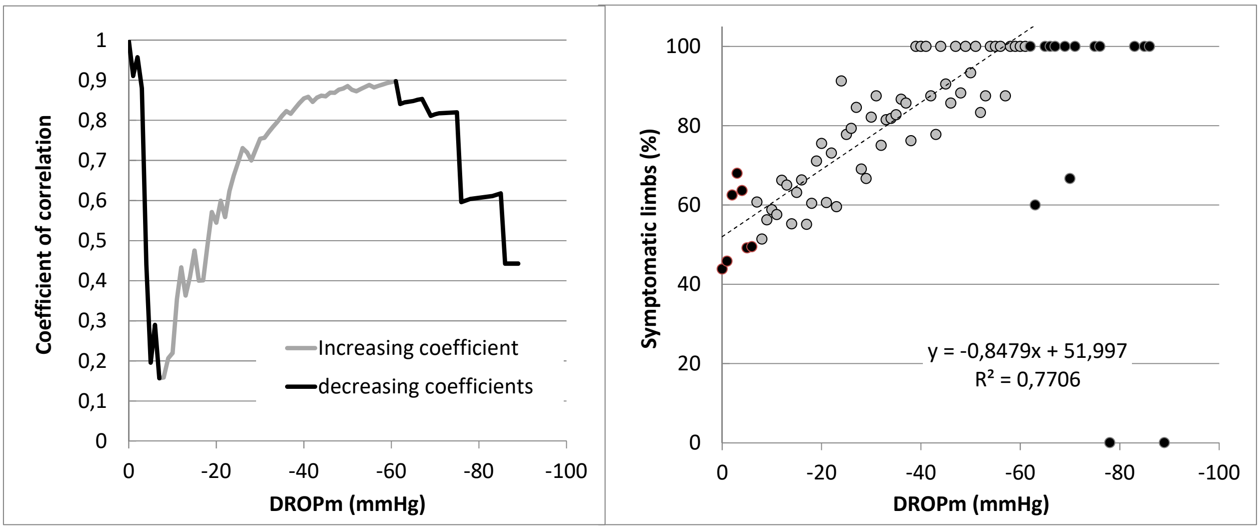

Figure S3: *Total number of observations (grey circles) and the resulting number of symptomatic cases likely resulting from arterial TOS (black squares) after application of the coefficient (Dash line) to symptomatic cases only for each unit of DROPm. The final number of symptomatic cases likely resulting from aTOS in the population is the sum of all cases in black squares.*

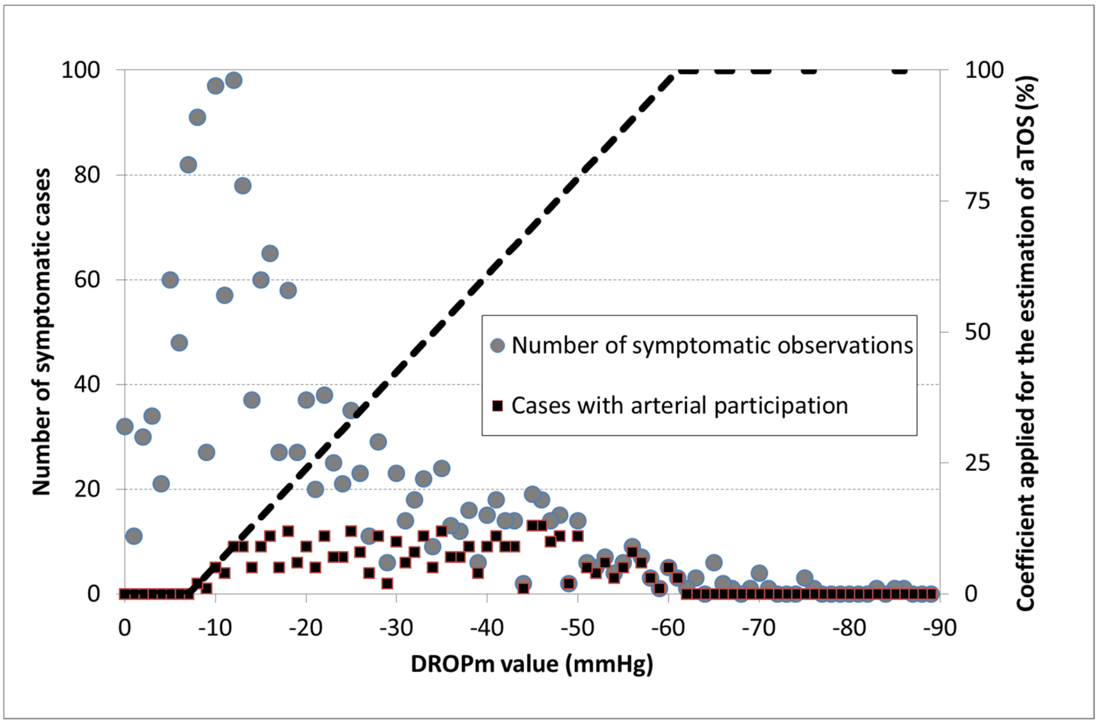

Supplement: Supplementary file 1 [file jcm-13-03925-s001.zip › jcm-3058017-supplementary.pdf]
